# Failure to report protocol violations in clinical trials: a threat to internal validity?

**DOI:** 10.1186/1745-6215-12-214

**Published:** 2011-09-28

**Authors:** Elizabeth A Sweetman, Gordon S Doig

**Affiliations:** 1 Northern Clinical School Intensive Care Research Unit, University of Sydney, Sydney, Australia; 2 Faculty of Medicine, University of Sydney, Sydney, Australia

**Keywords:** Clinical trials, methods, methodology, protocol violations, protocol deviations, errors of conduct, reporting artifact, randomized, randomised

## Abstract

**Background:**

Excessive protocol violations (PV), which can be defined as preventable mistakes in study conduct, may result in patient harm and introduce errors into a clinical trial's results leading to flawed trial conclusions.

The purpose of this project was to gain a better understanding of reported PVs, to describe current practice with regards to the use of methods for the reduction of PVs and to investigate relationships between clinical trial characteristics and PVs.

**Methods:**

We reviewed 80 clinical trials conducted across a broad range of medical specialties published in four major general medical journals (The Lancet, NEJM, JAMA, BMJ). Eligible papers were identified using a PubMed search. For each included trial, two authors independently abstracted information on trial characteristics, PV reporting and PV rates and interventions used to reduce PVs. PVs were categorised into one of five distinct types: enrolment, randomisation, study intervention, patient compliance and data collection errors. Associations between PVs and study characteristics were investigated using logistic regression.

**Results:**

Eighty clinical trials (20 from each journal) were identified from 101 consecutive PubMed abstracts. The median number of participants was 701 (range: 20 to 162, 367) and the median number of participating sites was 15 (range: 1 to 701). Nineteen percent (15/80) of included trials were single centre trials. The median study duration was 24 months (range: 5.81 - 127 months) and 74% (59/80) of included trials were primarily academic funded.

Thirty two percent (26/80) of included trials failed to provide explicit reporting of any type of PV and none (0/80) of the trials provided explicit reporting of all five types of PVs. Larger clinical trials (more patients, more sites, longer duration, more complex management structure) were more likely to have more complete reporting of PV's.

Only 9% (7/80) of trials reported the use of a specific study method to prevent PVs. Use of a run-in phase was the only method reported.

**Conclusions:**

PVs are under-reported. Although the CONSORT statement provides guidance on the reporting of PVs, reporting requirements are not explicit for all types of PVs. As a first step towards improved reporting by authors, we recommend the CONSORT statement highlight the importance of PVs by making reporting requirements more explicit.

## Background

Preventable errors in study conduct, also known as protocol violations (PV), may lead to the introduction of bias (systematic errors), play of chance (random errors), and design errors into a clinical trial that ultimately results in flawed conclusions [1]. For example, poor study conduct leading to errors in study treatment dosing may cause harm to enrolled patients [2]. Avoidable harm arising from incorrect dose calculations will dilute any true treatment benefits thus reducing overall statistical power leading to a false negative result [1,3]. An understanding of PV rates may therefore enhance the interpretation of a clinical trial's results.

There is no general agreement on what constitutes appropriate thresholds for acceptable and excessive PV rates. One authority on the conduct of clinical trials has suggested that PVs in more than 10% of enrolled patients is excessive and "reflect[s] a generally poor standard of trial organisation which needs tightening up" [3]. Post hoc evaluation committees analysing completed Food and Drug Administration (FDA) Phase III licensing trials have reported PVs ranging from 15.6% (88/564) [4] to 24.9% (431/1728) [2,5] of all enrolled patients, however neither committee classified these levels as excessive. We conducted an extensive literature search [6] to identify publications on this topic. Although we could find no reviews providing overall estimates of PV rates, much has been written about interventions for reducing PVs [1-3,6].

Studies have been conducted to demonstrate that different types of PVs can be prevented by using certain study interventions or design features. For instance, a *study run-in phase *can be used to identify patients for subsequent enrolment who are more likely to be compliant with study interventions, resulting in improved power due to reduced study treatment related PVs [7]. A study run-in phase also provides a protected learning environment such that researchers can become more familiar with study processes and procedures, leading to a reduction in other types of PVs such as enrolment errors [6]. Furthermore, enhanced educational interventions such as web based tutorials that provide feedback throughout the conduct of a trial can reduce data collection related PVs and study intervention PVs [1]. Despite the availability of methods proven to enhance trial conduct, we are uncertain as to how many trialists actually embrace these methods.

The purpose of this project was to gain a better understanding of reported PV rates, to describe current practice with regards to the use of methods for the reduction of PVs and to investigate relationships between clinical trial characteristics and PV rates. To achieve these goals we reviewed 80 consecutive clinical trials published in four major journals.

## Methods

### Primary Literature Search

To detect eligible clinical trials for this review we searched the Lancet, the New England Journal of Medicine, the Journal of the American Medical Association and the British Medical Journal using the National Library of Medicine's search engine *PubMed *http://www.PubMed.org. Phrases optimized to detect randomised controlled trials (RCTs) [8] were crossed with Medical Subject Heading (MeSH) Journal Titles. A complete list of the MeSH Journal Title searches is presented in Table 1.

**Table 1:** Journal name search terms used in primary MEDLINE search

Journal	Medical Subject Heading Terms
The Lancet	("Lancet"[Journal] OR "lancet"[All Fields])

New England Journal of Medicine	"N Engl J Med"[Journal]

Journal of the American Medical Association	("JAMA"[Journal] OR "jama"[All Fields])

British Medical Journal	("Br Med J"[Journal] OR "Br Med J (Clin Res Ed)"[Journal] OR "BMJ"[Journal] OR "bmj"[All Fields])

Consecutive clinical trials published after May 1^st ^2009 were retrieved.

### Study Selection

All identified abstracts were independently reviewed by both authors (EAS, GSD). The authors were not blinded to publication source or abstract author list. Any abstract that either author believed constituted a clinical trial was retrieved in full text for detailed review.

Primary publications of cluster randomised and individual patient randomised trials, evaluating any type of intervention (education, drug treatment, surgery etc) were eligible for inclusion. Publications of subgroup analyses or economic analyses from previously published trials were not eligible.

### Trial Characteristics

For each included clinical trial, the following trial characteristics were independently appraised and extracted by both authors (EAS, GSD): study type (individual patient randomised, cluster randomised or factorial design); total number of study arms; patient population (adults, children or infants); study intervention (drug, surgery, education, other); number of patients randomised; number of study sites; funding source for trial (primarily academic, primarily commercial); reporting of trial education/start-up processes; response to protocol violations; trial results (positive, neutral or harm); reporting of adherence to Good Clinical Practice (GCP) recommendations; lead investigator previous experience with research in the field (previous on topic clinical trial or observational study reported in reference list); risk of bias [9] including reporting of randomisation, blinding and, allocation concealment and; management structure (type of management structure e.g. management committee, steering committee etc.). PV rates and specific methods used to reduce or prevent future PVs were also abstracted from each paper.

Disagreements between the authors regarding study inclusion or appraisal were resolved by discussion.

### Types of Protocol Violations

Wolf and Makuch define a PV as a departure from the guidelines specified in the study protocol that could have been prevented by the investigator [10]. Under their classification scheme, an *unpreventable *departure from the study protocol is referred to as a *protocol deviation*. For example, discontinuation of the study intervention due to an unforeseen adverse event or an act of nature (Ex. Hurricane Katrina) would be classified a *protocol deviation*, not a PV. For the purposes of this study, we did not consider any departure from the study protocol to be a PV if the departure was considered necessary to protect the safety of a patient.

We identified and reported on five distinct types of PVs:

**1) *Enrolment PVs ***occurred when a member of the research team failed to appropriately apply the study's eligibility criteria resulting in the enrolment of an inappropriate patient into the trial.

**2) **A ***randomisation PV ***was defined as a technical or human error leading to the violation of the intended randomisation sequence or any attempts to subvert allocation concealment.

**3) **A ***study intervention PV ***was defined as a dosing, timing or delivery error in the study intervention attributable to members of the research team. The research team included members of the study coordinating centre, site investigators, research coordinators and members of the healthcare team caring for participants.

**4) **A ***patient compliance PV ***involved study participants failing to comply with the trial protocol regarding a study intervention or other requirements of participation in the trial (e.g. skipping scheduled appointments). Formal withdrawal of consent to participate was not considered a patient compliance PV.

**5) *Data collection PVs ***encompassed errors in which the research team failed to comply with pre-specific trial guidelines for data collection and/or outcome evaluation due to avoidable reasons.

The reporting of each type of PV was further described as *explicit*, *incomplete *or *absent*. *Explicit *reporting allowed complete categorisation of the PV by type and cause. Reporting of cause and type allowed the determination of whether the departure from protocol was preventable or safety related.

### Analysis

The frequency of occurrence of PVs is reported by *type *of PV, calculated as number of PVs divided by number of enrolled patients. Proportions were only calculated when PV reporting was explicit. If PVs were not reported, we did not infer that the PV rate was zero.

Relationships between study characteristics and PVs were investigated using chi-square tests, t-tests or logistic regression, depending on the independent variable. A two-tailed P-value less than 0.05 was accepted to demonstrate the presence of statistical significance.

## Results

### Primary Literature Search and Study Selection

Hand searching of identified abstracts resulted in the retrieval of 101 full text papers for detailed review. Eighty clinical trials, 20 consecutive trials from each major journal, were identified from these 101 retrieved papers. Reasons for exclusion of 21 papers included: the paper was a subgroup or economic analysis of a previously published clinical trial (6); the paper was not a clinical trial (4); the paper was not published in the target journal (4); systematic reviews (2) or; other reasons (5).

### Trial Characteristics and Risk of Bias

The median number of participants in the included clinical trials was 701 (range: 20 to 162, 367) and the median number of participating sites was 15 (range: 1 to 701). Nineteen percent (15/80) of included trials were single centre studies. The median study duration was 24 months (range: 5.81 - 127 months) and 74% (59/80) of included trials were primarily academic funded. Sixty five percent (52/80) of the trials reported a significant positive treatment effect, 32% (26/80) had neutral (negative) findings and 2.5% (2/80) reported significant harm. Complete characteristics of the included trials can be found in Table 2. Risk of bias is reported in Table 3.

**Table 2:** Characteristics of included trials

Trial Characteristics	Percent (number) of Trials
Study Type	
Individual patient RCT	76.25% (61/80)
Factorial RCT	6.25% (5/80)
Cluster RCT	17.5% (14/80)

Number of study arms	
Two	83.75% (67/80)
Three	7.5% (6/80)
Four	8.75% (7/80)

Patient Population	
Adults	91.25% (73/80)
Children	6.25% (5/80)
Infants	1.25% (1/80)
Mixed (Adults & Children)	1.25% (1/80)

Study Intervention	
Drug	46.25% (37/80)
Surgery	8.75% (7/80)
Education	10.0% (8/80)
Other	27.5% (22/80)
Procedure	3.75% (3/80)
Weight loss	3.75% (3/80)

Funding Source	
Academic	73.75% (59/80)
Industry	26.25% (21/80)

Trial start-up meeting described	8.75% YES (7/80)

Trial run-in phase described	8.75% YES (7/80)

GCP adherence reported	6.25% YES (5/80)

Lead Investigator reports previous research experience	62.5% YES (50/80)

Trial results	
Harm	2.5% (2/80)
Neutral	32.5% (26/80)
Positive	65.0% (52/80)

Management structure	
MC, SC & others (explicit structure)	26.25% (21/80)
MC or SC only (basic structure)	21.25% (17/80)
Not reported	52.8% (42/80)

Abbreviations: RCT - Randomised controlled trials; MC - Management Committee; SC - Steering Committee; GCP - Good Clinical Practice.

**Table 3:** Risk of bias of included trials

Methodological Characteristics	Percent (number) of Trials
Details regarding generation of the randomisation sequence	88.75% (71/80)

Reporting of Allocation Concealment	
Maintained	52.5% (42/80)
Unclear	45.0% (36/80)
Not maintained	2.5% (2/80)

Use of any type of blinding	58.75% (47/80)

Presentation of results according to ITT	71.25% (57/80)

Loss to follow-up reported by study arm	92.50% (74/80)

Abbreviations: ITT - Intention To Treat.

### Protocol Violation Reporting

Thirty-two percent (26/80) of included trials failed to provide *explicit reporting *of *any type *of PV. None (0/80) of the trials provided explicit reporting of all five types of PVs.

Explicit reporting varied widely according to PV type, with explicit reporting highest for patient compliance PVs (47.5%, 38/80 trials) and lowest for randomisation PVs (8.75%, 7/80 trials). Table 4 provides complete reporting details for all types of PVs.

**Table 4:** Protocol violation reporting and protocol violation frequency.

Protocol Violation Type	Percent (number) of trials	Proportion of Enrolled Participants with PVs Median (range) Calculated from trials with Explicit Reporting
Enrolment PVs		
Explicit reporting	12.5% (10/80)	0.8% (1.6% to 9.1%)
Incomplete reporting	8.8% (7/80)	
Absent	78.8% (63/80)	

Randomisation PVs		
Explicit reporting	8.8% (7/80)	0.7% (0.04% to 7.7%)
Incomplete reporting	1.3% (1/80)	
Absent	90.0% (72/80)	

Study Intervention PVs		
Explicit reporting	21.2% (17/80)	1.3% (0% to 13.2%)
Incomplete reporting	21.2% (17/80)	
Absent	57.5% (46/80)	

Patient compliance PVs		
Explicit reporting	47.5% (38/80)	7% (0.2% to 87%)
Incomplete reporting	25.0% (20/80)	
Absent	27.5% (22/80)	

Data Collection PVs		
Explicit reporting	21.3% (17/80)	1.7% (0.0% to 16.1%)
Incomplete reporting	18.8% (15/80)	
Absent	60.0% (48/80)	

Abbreviations: PV - protocol violations.

### Proportion of Enrolled Participants with Protocol Violations

Of the 38 trials with explicit reporting of patient compliance PVs, the median proportion of patients with compliance PVs was 7% (range 0.2% to 87%) of all enrolled patients. Thus, actual compliance was 93% (range: 13% to 99.8%). Table 4 reports PV occurrence by type, abstracted from trials with *explicit *reporting.

### Reporting of methods used to reduce or prevent PVs

Only 9% (7/80) of included trials reported *any *details regarding study start-up/site initiation meetings or other forms of educational training of the research team. Nine percent (7/80) trials reported the use of a run-in phase. No other methods were mentioned.

### Relationships between trial characteristics and PV reporting

#### Enrolment PVs

As study duration increased, reporting of enrolment PVs increased (P_LR chi-square _< 0.0001, OR = 1.053, 95% CI 1.022 to 1.085) and studies reporting any form of management structure were more likely to report enrolment PVs (P_LR chi-square _= 0.0221, 26/42 management structure not reported vs. 16/17 basic vs 16/21 more detailed).

Studies that presented ITT results were less likely to report enrolment PVs (P_Exact _= 0.0313 8/57 vs 9/23) and there was a trend for studies that reported details of the generation of randomisation sequence to be less likely to report enrolment PVs (P_Exact _= 0.0903, 13/71 vs 4/9)

#### Randomisation PVs

As number of sites increased, a study was more likely to report randomisation PVs (P_LR chi-square _= 0.0662, OR = 1.004, 95% CI 1.000 to 1.008) and industry funded trials were more likely to report randomisation PVs (P_Exact _= 0.0263, 5/21 Industry vs 3/59).

#### Study Intervention PVs

There was a significant difference (P _LR chi-square _= 0.0330) in study intervention PV reporting between the major journals (7/20 BMJ vs. 4/20 JAMA vs. 11/20 Lancet vs. 12/20 NEJM).

As study duration increased, study intervention PV reporting became more likely (P_LR chi-square _= 0.0500, OR = 1.021 per month, 95% CI 1.000 to 1.042) and studies reporting any form of management structure were more likely to report study intervention PVs (P_LR chi-square _= 0.0500, 12/42 management structure not reported vs. 11/17 basic reporting vs 11/21 detailed reporting). Study intervention PV reporting also became more likely as number of study sites increased (P_LR chi-square _= 0.0184, OR = 1.006 per study site, 95% CI 1.001 to 1.020).

#### Patient Compliance PVs

There were no significant relationships between any trial characteristics and the reporting of patient compliance PVs.

#### Data collection PVs

As study duration increased, papers were more likely to report data collection PVs (P_LR chi-square _= 0.0524, OR 1.021, 95% CI 1.000 to 1.042) however studies reporting ITT results were *less *likely to report data collection PVs (P _Exact _= 0.0777, 19/57 ITT vs 13/23 no ITT). Studies reporting adherence to GCP recommendations were also *less *likely to report data collection PVs (P _Exact _= 0.0712, 0/5 GCP reported vs. 32/75 GCP not reported).

## Discussion

We conducted a comprehensive review of 80 clinical trials published in four major medical journals in order to gain a better understanding of reported PV rates, to describe current practice with regards to the use of methods for the reduction of PVs and to investigate relationships between clinical trial characteristics and PV rates. Overall, we found that PV reporting was poor.

One third of all reviewed trials failed to provide reporting of any types of PVs. None of the reviewed trials presented complete reporting for all types of PVs. We found that larger clinical trials (more patients enrolled, more study sites, longer duration of recruitment, more complex management structures) were likely to have more complete reporting of PVs.

### PV reporting

The original and revised CONSORT statements recommend that departures from the study protocol should be reported [11,12]. Reporting of PVs is essential when establishing the appropriateness of excluding patients from a modified intention to treat analysis or an efficacy subset (per-protocol) analysis [13,14]. Because excessive PVs have been linked to patient harms [2], which may dilute the benefits attributable to a truly effective treatment leading to false negative clinical trials results [4], reporting of protocol errors may also be essential for the interpretation of negative trial results. Furthermore, full reporting of PVs is vital to the design of post-approval safety assessments of new interventions [15]. Despite the plethora of reasons to motivate authors to report these important measures of good study conduct, we found reporting of PVs was uncommon.

### PV frequency

Patient compliance PVs were the most commonly reported type of PV and were documented explicitly in 48% of trials. The next most commonly reported were study intervention PVs (21%), data collection PVs (21%), enrolment PVs (12%) and technical randomisation PVs (9%). Estimates of within-trial PV rates obtained from this review may be biased due to poor reporting because, as with other aspects of trial conduct, an absence of PV reporting could not be assumed to infer no PVs were experienced [16].

Of the 54 clinical trials that did provide explicit reporting of PV rates, 29% (16/54) reported a PV rate greater than the 10% threshold defined as *excessive *by Pocock [3]. Even in the face of incomplete reporting, this finding suggests there is room for improvement. It is likely that a PV rate below 10% could be achieved in each of these 16 trials if conduct was improved.

### Preventing PVs

In order to improve trial conduct, PVs need to be identified *during *a clinical trial such that they can be studied and unnecessary errors can be prevented from occurring again [3]. For example, when a PV is detected during the conduct of a multi-centre trial, the trial's clinical coordinating centre (CCC) may provide immediate non-punitive positive feedback and education to all study sites to minimise the chances of the same PV occurring again [6]. None of the reviewed clinical trials explicitly reported how PVs were studied and prevented during trial conduct however seven of the reviewed trials did report use of a run-in phase.

Prior to recruitment into a trial, a *run-in phase *may be conducted to identify noncompliant participants and exclude them from subsequent study enrolment [17]. During a run-in phase, potential participants are given a test exercise to complete, such as taking 10 days of placebo medication. Patients are graded on their performance of this test exercise and only patients who are considered to be *compliant *with the exercise are enrolled and randomised into the trial [17].

Furthermore, if other processes and procedures are *simulated *during a run-in phase, other types of PVs can be minimized [6].

By preventing enrolment of the least compliant patients, it is accepted that a successful run-in phase can increase the overall power of a study however, compared to results that would have been observed without a run-in phase, the reported results may overestimate the benefits and underestimate the risks of treatment, underestimate the number needed to treat, and yield a smaller P-value [17]. These considerations must be balanced against the desire to minimise false negative results by enrolling only those patients who will be compliant with the study intervention.

### Factors related to improved reporting

To the best of our knowledge, our investigation of trial characteristics associated with PV reporting was the first time such a question has been addressed. Our initial intent was to conduct an investigation into trial characteristics associated with PV *rates*, however with such poor reporting of PVs, we were not able to conduct this analysis. Even with the best reported type of PV (patient compliance), up to 50% of trials failed to report a *rate*. With a best case scenario of 50% missing data, we were concerned that estimates of PV rates were open to significant potential biases and that any analysis of trial characteristics associated with PV rates would be under-powered and of questionable veracity. We therefore focused our analysis on an investigation of trial characteristics and PV *reporting *(reported Yes or No).

In general, our analysis indicated that trials with characteristics common to 'larger' studies (more patients, more sites, longer duration, more complex management structure) were more likely to report PVs. It is possible that larger studies are more complex to coordinate [18] and therefore may have *more *PVs which leads to more frequent reporting of PVs. It is also possible however, that the improved management structures associated with increasing trial size facilitates the recording and reporting of PVs, regardless of PV rate. We strongly recommend more research into this issue however reporting of PV rates must be improved first.

Finally, it is important to acknowledge that we did identify a significant difference in study intervention PV reporting between journals. Inspection of the two journals with the lowest intervention PV reporting (BMJ, JAMA) revealed that they tended to publish smaller trials (shorter duration, fewer study sites) that were less likely to report any form of management structure. It is possible the difference in intervention PV reporting observed between journals is attributable to differences in trial characteristics as opposed to journal policies. Future studies are needed to investigate this finding in more detail.

### Recommendations

Whilst the CONSORT statement does recommend reporting of PVs [11,12] the format does not place a heavy emphasis on this issue. We believe the CONSORT statement should highlight the importance of PVs by making reporting requirements more explicit. It is accepted in the literature that authors and journal editors *do *respond to CONSORT recommendations by improving reporting of trial features [19,20]. Modifications to the CONSORT statement are the first step towards improved reporting of PVs.

Clinical trialists should embrace Wolf's definition of a protocol violation [10] because it incorporates the concept of *causality *which allows a trialist to identify PVs that are *preventable*. Application of this definition to identify errors in conduct that occur early during a trial may allow future errors to be avoided, thus reducing overall PV rates.

We recommend that journal editors should require full reporting of PVs. Full reporting will enable future research projects to identify relationships between PVs and study characteristics and to examine any influence of excessive PVs on study results. Research in this field may lead to improvements in the overall conduct of future clinical trials.

### Strengths and Weaknesses

To the best of our knowledge, this study is the first review to describe PV reporting in a sample of clinical trials which were conducted across a broad range of medical specialties and published in four major general medical journals. Others have based methodological reviews on these four major journals [21-24] however clinical trials published in these journals may not be representative of all published trials.

Compared to those published in specialty journals, clinical trials published in The Lancet, NEJM, JAMA and the BMJ are known to be larger (more patients, more study sites, longer duration of recruitment) and are more likely to be regarded as low risk of bias due to improved reporting of methodological details [25]. Because the findings of this report are based on a very select type of *atypical *trial, it is possible the current results do not generalise to trials published in other journals. Given we found poor reporting of PVs in these major general medical journals, there is no reason to suspect that PV reporting is better in sub-specialist journals however, more research is required to address this issue.

The published literature provides many working definitions of a protocol violation or protocol deviation. We chose the definition by Wolf [10] because it encompasses the concept of causality which helps identify study errors that are preventable [3]. Wolf defines a PV as a preventable error in study conduct, whereas a protocol deviation is unpreventable (e.g. study drug must be stopped due to safety etc.). Furthermore, we classified PVs into five main types based on the study process involved and source of the error. We find this classification useful because it aids in the identification of an appropriate response to the error.

Another limitation of this study is the relatively small sample size of 80 papers. It is possible that we failed to find some significant associations between trial characteristics and PV reporting due to sub-optimal power. Although we found interesting associations, larger studies may be required to investigate PVs in more detail.

## Conclusions

We undertook this comprehensive review to gain a better understanding of reported PV rates, to describe current practice with regards to the use of methods for the reduction of PVs and to investigate relationships between clinical trial characteristics and PV rates.

Overall, we found that reporting of PVs was poor however larger trials were more likely to report PVs. Furthermore, few clinical trials documented methods used to prevent or minimise PVs.

Preventable errors in study conduct may lead to avoidable patient harm and may result in false negative trial results. As a first step towards improving our understanding of the influence of PVs, reporting must be improved. We recommend changes to the CONSORT statement that make reporting requirements for PVs more explicit. Once reporting is improved, more research may reveal how to prevent and minimize PVs. For example, a severity grading system for PVs may allow clinical trialists to focus on preventing the most important types of PV thereby reducing avoidable patient harm and removing bias from the results of our clinical trials.

## List of Abbreviations Used

95% CI: 95% Confidence Interval; OR: Odds Ratio; PV: Protocol Violation; RCT: Randomised Controlled Trial; CCC: Clinical Coordinating Centre; ITT: Intention To Treat.

## Competing interests

The authors declare that they have no competing interests.

## Authors' contributions

EAS and GSD conceived and designed the study, managed the project, conducted the statistical analysis, interpreted the results and drafted the manuscript. GSD had full access to all of the data in the study and takes full responsibility for the integrity of the data and the accuracy of the data analysis.

## Author details

EAS is a Senior Research Fellow at the Northern Clinical School Intensive Care Research Unit, University of Sydney, Sydney, Australia. GSD is the Head of the Northern Clinical School Intensive Care Research Unit, University of Sydney, Sydney, Australia and Associate Professor in Intensive Care, at the Faculty of Medicine, University of Sydney, Sydney, Australia.
